# Multidimensional psychosocial screening in routine cardiac care: preliminary findings from a cross-sectional survey

**DOI:** 10.1007/s00059-026-05382-w

**Published:** 2026-06-24

**Authors:** Omar Hahad, Jasmin Ghaemi Kerahrodi, Leila I. Pasha, Michael Molitor, Matthias Michal, Jonas Tesarz, Philipp Lurz, Julia Lurz

**Affiliations:** 1 Department of Cardiology, Cardiology I, University Medical Center of the Johannes Gutenberg-University Mainz, Langenbeckstraße 1, 55131 Mainz, Germany; 2 Partner Site Rhine-Main, German Center for Cardiovascular Research (DZHK), Mainz, Germany; 3 Department of Psychosomatic Medicine and Psychotherapy, University Medical Center of the Johannes Gutenberg-University Mainz, Mainz, Germany; 4 Cardioangiologisches Centrum Bethanien (CCB), Agaplesion Markus-Krankenhaus, Frankfurt am Main, Germany

**Keywords:** Psychocardiology, Cardiovascular disease, Heart-focused anxiety, Sleep disturbances, Mental health integration, Psychokardiologie, Herz-Kreislauf-Erkrankungen, Herzbezogene Angst, Schlafstörungen, Integration psychischer Gesundheit

## Abstract

**Background:**

Psychological distress is common among patients with cardiovascular disease. While European Society of Cardiology (ESC) position papers highlight the relevance of mental health in cardiology care, psychosocial assessment remains inconsistently implemented in routine clinical practice.

**Methods:**

In this preliminary cross-sectional survey, patients attending a tertiary cardiology department completed an anonymous multidimensional psychosocial screening during routine care. Standardized instruments assessed depressive symptoms (PHQ-9), generalized anxiety (GAD-7), heart-focused anxiety (HAF-17), sleep disturbances (Jenkins Sleep Scale), resilient coping (BRCS), loneliness (UCLA-3), life satisfaction, perceived stress, social support, medication adherence, and awareness of psychocardiology services.

**Results:**

A total of 62 patients were included. Overall levels of depressive and generalized anxiety symptoms were low (PHQ‑9 5.0 ± 4.7; GAD‑7 3.5 ± 4.4), while heart-focused anxiety was more pronounced (HAF-17 21.1 ± 10.6). Perceived stress was reported by 52.1% of participants (43.8% mild, 8.3% severe), and sleep disturbances were frequent (JSS 6.5 ± 5.5). Heart-focused anxiety showed consistent associations with general anxiety, depressive symptoms, stress, and sleep problems (all *p* < 0.05), but was not related to life satisfaction (*p* = 0.99). Life satisfaction correlated more strongly with general emotional well-being than with cardiac-specific fears. Despite limited prior awareness of psychocardiology services (7.0% aware), a substantial proportion of patients reported interest in receiving psychosocial support in a cardiology setting (8.8% yes and 47.4% maybe).

**Conclusion:**

Psychosocial burden in cardiology patients may extend beyond depression and generalized anxiety. Cardiac-specific fears, stress, and sleep disturbances are prominent but frequently underrecognized domains that may be overlooked by unidimensional screening approaches. A significant proportion of patients were unaware of existing psychocardiology services, suggesting a gap between recommended mental health integration and routine cardiology care. Multidimensional screening may facilitate more targeted identification of patients who could benefit from integrated psychocardiological care.

## Abstract (de)

**Hintergrund:**

Psychische Belastungen sind bei Patienten mit Herz-Kreislauf-Erkrankungen weit verbreitet. Obwohl die Stellungnahmen der Europäischen Gesellschaft für Kardiologie (ESC) die Bedeutung der psychischen Gesundheit in der kardiologischen Versorgung hervorheben, wird die psychosoziale Beurteilung in der klinischen Routinepraxis nach wie vor nicht einheitlich umgesetzt.

**Methoden:**

In dieser vorläufigen Querschnittsstudie füllten Patienten, die eine kardiologische Fachklinik aufsuchten, im Rahmen der Routineversorgung einen anonymen, mehrdimensionalen psychosozialen Fragebogen aus. Mit standardisierten Instrumenten wurden depressive Symptome (PHQ-9), generalisierte Angst (GAD-7), herzbezogene Angst (HAF-17), Schlafstörungen (Jenkins Sleep Scale), resiliente Bewältigungsstrategien (BRCS), Einsamkeit (UCLA-3), Lebenszufriedenheit, wahrgenommener Stress, soziale Unterstützung, Medikamenteneinnahme und das Wissen um psychokardiologische Dienstleistungen erfasst.

**Ergebnisse:**

Insgesamt wurden 62 Patienten in die Studie aufgenommen. Das allgemeine Ausmaß an depressiven Symptomen und generalisierten Angstsymptomen war gering (PHQ‑9 5,0 ± 4,7; GAD‑7 3,5 ± 4,4), während die herzbezogene Angst stärker ausgeprägt war (HAF-17 21,1 ± 10,6). 52,1 % der Teilnehmenden gaben an, Stress zu empfinden (43,8 % leicht; 8,3 % schwer), und Schlafstörungen traten häufig auf (JSS 6,5 ± 5,5). Herzbezogene Angst zeigte konsistente Zusammenhänge mit allgemeiner Angst, depressiven Symptomen, Stress und Schlafproblemen (alle *p* < 0,05), stand jedoch in keinem Zusammenhang mit der Lebenszufriedenheit (*p* = 0,99). Die Lebenszufriedenheit korrelierte stärker mit dem allgemeinen emotionalen Wohlbefinden als mit herzspezifischen Ängsten. Trotz mangelnder Kenntnis über psychokardiologische Angebote (7,0 % wussten um diese) zeigte ein erheblicher Anteil der Patienten Interesse an psychosozialer Unterstützung in einer kardiologischen Einrichtung (8,8 % „ja“ und 47,4 % „vielleicht“).

**Schlussfolgerung:**

Die psychosoziale Belastung bei kardiologischen Patienten kann über Depressionen und generalisierte Angstzustände hinausgehen. Herzspezifische Ängste, Stress und Schlafstörungen sind wichtige, jedoch häufig unterschätzte Aspekte, die bei eindimensionalen Screening-Ansätzen leicht übersehen werden können. Ein erheblicher Anteil der Patienten wusste nichts von bestehenden psychokardiologischen Angeboten, was auf eine Lücke zwischen der empfohlenen Integration psychischer Gesundheitsversorgung und der routinemäßigen kardiologischen Versorgung hindeutet. Ein mehrdimensionales Screening könnte eine gezieltere Identifizierung von Patienten ermöglichen, die von einer integrierten psychokardiologischen Versorgung profitieren könnten.

Cardiovascular and mental health are closely linked, yet their integration into routine cardiology care remains insufficient [1]. Psychological distress is highly prevalent among patients with cardiovascular disease and is associated with reduced quality of life [2], poorer treatment adherence [3], and increased morbidity and mortality [4–6].

Depressive and anxiety symptoms are the most extensively studied psychosocial factors in cardiovascular populations [7]. Both are associated with worse prognosis across a broad spectrum of cardiovascular conditions, including coronary artery disease [8], heart failure [9], and cardiac arrhythmias [10]. Several mechanisms have been suggested to explain these links, such as genetic predisposition, autonomic imbalance, chronic low-grade inflammation, dysregulation of neuroendocrine stress systems, and adverse health behaviors [11, 12]. Importantly, interventional evidence supports the clinical relevance of mental health in cardiology. In the randomized MENTAL AF trial, Lurz et al. demonstrated that an app-based mental training initiated after pulmonary vein isolation significantly reduced atrial fibrillation-related symptom burden and improved quality of life compared with standard care, highlighting the potential of psychological interventions as adjunctive strategies in arrhythmia management [13]. Consistent with this, the TEACH study highlighted both the feasibility and clinical relevance of systematic psychosocial screening in cardiology settings, while also pointing to substantial barriers to its routine implementation [14]. Together, these findings support the view that mental health is not simply a comorbidity but a relevant component of cardiovascular disease management.

This perspective is increasingly reflected in emerging scientific and clinical statements. In 2021, The American Heart Association [1] emphasized the importance of the mind–heart–body connection and called for greater attention to psychological health in cardiovascular care [15]. More recently, the 2025 European Society of Cardiology (ESC) Clinical Consensus Statement on mental health and cardiovascular disease provided practical recommendations for implementing mental health screening in routine cardiology practice including multidisciplinary psychocardiological care models [15, 16]. The ESC document highlights the Patient Health Questionnaire (PHQ; [17]) and the Generalized Anxiety Disorder (GAD) scale [18] as feasible questionnaires to identify patients with clinically relevant depressive and anxiety symptoms.

At the same time, depression and generalized anxiety capture only part of the psychosocial burden experienced by cardiac patients. Other dimensions, such as heart-focused anxiety [19], sleep disturbances [20], and loneliness [21, 22], are common in cardiac populations and have been associated with heightened symptom perception, poorer health behaviors, and higher healthcare utilization.

Against this background, this study aims to provide an initial and exploratory characterization of psychosocial burden in a routine cardiology care setting using a multidimensional screening approach. Specifically, we sought to (a) describe the prevalence and distribution of psychosocial symptoms and resources; (b) examine the interrelationships between depressive symptoms, anxiety, heart-focused anxiety, sleep disturbances, loneliness, resilient coping, and life satisfaction; and (c) assess patient awareness of and interest in psychosocial support within cardiology care. By aligning with current ESC recommendations while extending the scope beyond depression and anxiety alone, this study aims to inform the implementation and further development of psychosocial screening strategies in routine cardiology practice.

## Methods

### Study design and setting

This cross-sectional survey study was conducted at the Department of Cardiology, Cardiology I at the University Medical Center of the Johannes Gutenberg University of Mainz, Germany. Data collection took place during November and December 2025 as part of routine clinical care. A consecutive sample of patients attending outpatient or inpatient cardiology care were invited to participate. The survey was administered in paper form and completed anonymously. Participation was voluntary, and non-participation had no consequences for medical treatment.

According to local regulations and institutional policies, fully anonymous questionnaire-based surveys that do not involve the collection of identifiable personal data do not require formal ethics committee approval. No personal identifiers were collected, and responses could not be linked to individual patients. The study was conducted in accordance with the Declaration of Helsinki. Participants received written information explaining the purpose and anonymous nature of the survey. Completion and return of the questionnaire were considered to imply informed consent. Because of the fully anonymous design, no individual clinical follow-up based on questionnaire responses was possible. Participants were informed that the survey did not replace medical or psychological care. Due to the fully anonymous design and non-tracked distribution process, the exact number of patients approached during the study period could not be reliably determined. The final sample therefore represents all returned and evaluable questionnaires.

### Data collection procedure

German versions of the questionnaires were distributed by clinical staff during routine cardiology visits or hospital stays. Patients completed the questionnaires independently and returned them to sealed collection boxes on the ward or in the outpatient clinic to ensure anonymity. The timing of questionnaire completion was not standardized and depended on the clinical workflow, resulting in variability with regard to time since admission or presentation.

### Measures

#### Sociodemographic and clinical information

Participants reported age, sex, height, weight, marital status, and employment status. Self-reported medical history included known heart disease, prior psychiatric diagnoses, past or current psychotherapy or psychiatric treatment, and psychopharmacological medication use.

#### Depressive symptoms (PHQ-9)

Depressive symptoms over the preceding 2 weeks were assessed using the PHQ‑9, a widely validated self-report instrument [23]. Each of the nine items is scored from 0 (“not at all”) to 3 (“nearly every day”), yielding a total score range of 0–27. Established cut-offs are 5 (mild), 10 (moderate), 15 (moderately severe), and 20 (severe) depression. The PHQ‑9 demonstrates good internal consistency (Cronbach’s α ≈ 0.86–0.89) and criterion validity in patients in general medical settings.

#### Anxiety symptoms (GAD-7)

Generalized anxiety symptoms were assessed with the seven-item Generalized Anxiety Disorder Scale (GAD‑7; [24]). Seven items are rated from 0 to 3, yielding a total score of 0–21. Cut-offs of 5, 10, and 15 represent mild, moderate, and severe anxiety, respectively. The GAD‑7 shows strong reliability (α ≈ 0.89) and good validity across clinical and somatic patient samples.

#### Heart-focused anxiety (HAF-17)

Heart-focused anxiety was assessed using the 17-item Heart Anxiety Questionnaire (HAF-17). Items are rated on a scale from 0 to 4 (“never” to “always”) yielding a total score range of 0–68, with higher scores indicating greater heart-focused anxiety. The instrument comprises the subscales fear, avoidance, and attention to cardiac sensations. The HAF-17 demonstrates acceptable to excellent internal consistency (Cronbach’s α = 0.69–0.92) and evidence of construct and convergent validity in cardiac and psychosomatic samples [25, 26].

#### Perceived stress

Perceived stress over the past 4 weeks was assessed using a single global item with three response categories (not impaired, mildly impaired, severely impaired). Single-item stress measures are commonly used in clinical screening contexts. Although multi-item scales such as the Perceived Stress Scale (PSS-10; [27]) are the psychometric standard, single-item stress measures have been shown to correlate meaningfully with validated multi-item instruments and to possess satisfactory validity for group-level analyses [28].

#### Sleep disturbances (JSS)

Sleep disturbances were assessed using the four-item Jenkins Sleep Scale (JSS), which measures the frequency of common sleep problems over the past month, including difficulties initiating sleep, nocturnal awakenings, early awakening, and non-restorative sleep. Items are rated on a frequency scale from 0 to 5 (0 = “not at all” to 5 = “22–31 days”), yielding a total score range of 0–20, with higher scores indicating greater sleep disturbance [29]. The JSS has demonstrated acceptable internal consistency (Cronbach’s α = 0.80; [30]).

#### Resilient coping (BRCS)

Adaptive coping tendencies were measured with the Brief Resilient Coping Scale (BRCS), a four-item scale scored from 1 to 5 per item (total score 4–20; [31]). A common interpretative categorization of scores is as follows: 4–13 (low resilient coping), 14–16 (medium resilient coping), and 17–20 (high resilient coping). The BRCS has demonstrated acceptable internal consistency (Cronbach’s α ≈ 0.78; [32]).

#### Social support

Perceived social support was assessed using a single global item asking whether participants had people they could rely on. Response categories were “no,” “mostly no,” “mostly yes,” and “yes, definitely.”

#### Life satisfaction

Overall life satisfaction was assessed using a single 11-point numeric rating scale ranging from 0 (“very dissatisfied”) to 10 (“very satisfied”), consistent with widely used life satisfaction/evaluation measures in large population studies [33].

#### Loneliness (UCLA-3)

Loneliness was assessed using the three-item short form of the UCLA Loneliness Scale, measuring feelings of lacking companionship, feeling left out, and feeling isolated from others. Items were rated on a three-point scale (1 = “hardly ever/never,” 2 = “some of the time,” 3 = “often”), yielding a total score range of 3–9, with higher scores indicating greater loneliness. The UCLA‑3 has demonstrated acceptable internal consistency and good convergent validity with the full UCLA Loneliness Scale in population-based studies [34].

#### Medication adherence

Medication adherence was assessed using a single self-report item asking how often participants took their prescribed medications as directed over the past 7 days. Response options ranged from “always” to “rarely/never” on a four-point scale (1 = “always,” 2 = “mostly,” 3 = “sometimes,” 4 = “rarely/never”), with higher values indicating lower adherence. A similar single-item question has been shown to predict clinically relevant outcomes such as hospitalization and death in patients with heart failure, supporting its use as a pragmatic screening measure in clinical research settings [35].

#### Psychocardiology awareness and interest

Participants were asked whether they had previously heard of psychocardiology and whether they would consider using a psychocardiological service (“yes”/“maybe”/“no”). These items were developed for this survey to assess awareness and perceived need.

### Statistical analysis

Descriptive statistics were calculated for all variables. Continuous variables are presented as means ± standard deviations or medians where appropriate and categorical variables are shown as frequencies and percentages. Scale scores were calculated according to standard scoring procedures.

Associations between psychosocial variables were examined using Spearman rank correlations due to non-normal score distributions. Correlation coefficients (ρ) with two-tailed *p* values are reported. Missing data were handled using available-case analysis. The number of valid observations (*n*) is reported for each variable. Statistical significance was defined as *p* < 0.05. Analyses were conducted using SPSS (version 31.0.0, IBM, Armonk, NY, USA).

## Results

### Study sample and clinical characteristics

A total of 62 patients undergoing routine cardiology care were included. The number of valid responses varied across variables due to item-level non-response and is reported accordingly in the tables. The sample was predominantly older with 69.3% aged 61 years or older. Most participants were male (61.4%), and the majority were married (59.7%). Nearly two thirds were retired (60.7%), while 31.1% were currently employed. Mean body height was 171.4 ± 11.9 cm and mean body weight was 80.5 ± 22.5 kg (body mass index 27.28 ± 7.77; Table 1).

**Table 1:** Sociodemographic characteristics.

Variable	*n* (%)/value
*Age (years)*	
31–45	1 (1.6)
46–60	17 (27.4)
61–75	25 (40.3)
> 75	19 (30.6)
Valid/missing *n*	62/0
*Sex*	
Male	35 (61.4)
Female	22 (38.6)
Valid/missing *n*	57/5
*Relationship status*	
Married	37 (59.7)
Separated/divorced	10 (16.1)
Widowed	6 (9.7)
In a partnership	4 (6.5)
Single	5 (8.1)
Valid/missing *n*	62/0
*Occupational status*	
Retired	37 (60.7)
Employed (incl. part-time)	19 (31.1)
Unemployed/sick leave	5 (8.2)
Valid/missing *n*	61/1
*Anthropometry*	
Body height [cm], mean (SD)	171.36 (11.91)
Valid/missing *n*	61/1
Body weight [kg], mean (SD)	80.48 (22.5)
Valid/missing *n*	62/0
Body mass index, mean (SD)	27.28 (7.77)
Valid/missing *n*	61/1

Percentages are based on valid responses.

A known cardiac disease was present in 77.0% of participants. The vast majority reported no history of a diagnosed psychiatric disorder (91.7%) and no current or previous psychotherapeutic or psychiatric treatment (91.7%). Psychopharmacological medication use was reported by 16.1% of patients (Table 2).

**Table 2:** Cardiac and psychiatric history.

Variable	*n* (%)
*Known heart disease*	
Yes	47 (77.0)
No	14 (23.0)
Valid/missing *n*	61/1
*Psychiatric condition ever diagnosed*	
No	55 (91.7)
Yes (current or past)	5 (8.3)
Valid/missing *n*	60/2
*Psychotherapeutic/psychiatric treatment*	
No	55 (91.7)
Yes, in the past	5 (8.3)
Valid/missing *n*	60/2
*Psychopharmacological medication*	
No	52 (83.9)
Yes	10 (16.1)
Valid/missing *n*	62/0

Percentages are based on valid responses.

### Mental health burden and psychosocial characteristics

Overall levels of depressive and anxiety symptoms were low to mild. The mean PHQ‑9 score was 5.0 ± 4.7 (median 3.0), and the mean GAD‑7 score was 3.5 ± 4.4 (median 2.0). Heart-focused anxiety, assessed using the HAF-17, showed a mean score of 21.1 ± 10.6 (median 20.0; Table 3).

**Table 3:** Mental health variables.

Variable	Value
*Depressive symptoms (PHQ‑9, 0–27)*	
Mean (SD)	5.0 (4.72)
Median	3.0
Valid/missing *n*	59/3
*Anxiety symptoms (GAD‑7, 0–21)*	
Mean (SD)	3.5 (4.42)
Median	2.0
Valid/missing *n*	58/4
*Heart-focused anxiety (HAF-17, 0–68)*	
Mean (SD)	21.14 (10.57)
Median	20.0
Valid/missing *n*	57/5

Percentages are based on valid responses.

*GAD‑7* Generalized Anxiety Disorder scale (7-item), *HAF-17* Heart Anxiety Questionnaire (17-item), *PHQ‑9* Patient Health Questionnaire (9-item).

A substantial proportion of participants reported some perceived stress during the preceding 4 weeks, with 43.8% describing mild impairment and 8.3% severe impairment. Sleep disturbances were common, with a mean JSS score of 6.5 ± 5.5. The mean loneliness score (UCLA-3) was 3.8 ± 1.3, while overall life satisfaction was relatively high (mean 7.2 ± 2.8). Most patients reported reliable social support (78.9%) and high medication adherence, with 87.5% indicating that they always took their medication as prescribed (Table 4).

**Table 4:** Stress, loneliness, sleep, and psychosocial resources.

Variable	*n* (%)/value
*Perceived stress (last 4 weeks)*	
Not impaired	23 (47.9)
Mildly impaired	21 (43.8)
Severely impaired	4 (8.3)
Valid/missing *n*	48/14
*Sleep disturbances (JSS, 0–20)*	
Mean (SD)	6.53 (5.51)
Median	6.0
Valid/missing *n*	56/6
*Resilient coping (BRCS, 4–20)*	
Mean (SD)	11.74 (4.60)
Median	12.0
Valid/missing n	54/8
*Loneliness (UCLA‑3, 3–9)*	
Mean (SD)	3.76 (1.28)
Median	3.0
Valid/missing *n*	55/7
*Social support*	
Yes, definitely	45 (78.9)
Less/none	12 (21.1)
Valid/missing *n*	57/5
*Life satisfaction (0–10)*	
Mean (SD)	7.18 (2.75)
Median	8.0
Valid/missing *n*	56/6
*Medication adherence*	
Always	49 (87.5)
Mostly	6 (10.7)
Sometimes	1 (1.8)
Valid/missing *n*	56/6

Percentages are based on valid responses.

*BRCS* Brief Resilient Coping Scale, *JSS* Jenkins Sleep Scale, *UCLA* UCLA Loneliness Scale

### Awareness of psychocardiology and interest in services

Only a small proportion of participants had previously heard of psychocardiology (7.0%). Nevertheless, more than half expressed potential interest in a psychocardiological service, with 8.8% responding “yes” and 47.4% responding “maybe” (Table 5).

**Table 5:** Psychocardiology awareness and interest.

Variable	*n* (%)
*Have you ever heard of psychocardiology?*	
No	53 (93.0)
Yes	4 (7.0)
Valid/missing *n*	57/5
*Would you like to make use of a psychocardiological service?*	
Yes	5 (8.8)
Maybe	27 (47.4)
No	25 (43.9)
Valid/missing *n*	57/5

Percentages are based on valid responses.

### Associations between psychosocial variables

Spearman correlation analyses revealed a strong association between depressive and anxiety symptoms (ρ = 0.763, *p* < 0.001). Heart-focused anxiety was moderately correlated with both depressive symptoms (ρ = 0.513, *p* < 0.001) and anxiety symptoms (ρ = 0.466, *p* < 0.001). In addition, heart-focused anxiety showed significant associations with sleep disturbances (ρ = 0.306, *p* = 0.022) and loneliness (ρ = 0.282, *p* = 0.037).

Sleep disturbances were positively associated with depressive symptoms (ρ = 0.431, *p* = 0.001) and anxiety symptoms (ρ = 0.316, *p* = 0.019). Loneliness was related to depressive symptoms (ρ = 0.334, *p* = 0.014) and anxiety symptoms (ρ = 0.365, *p* = 0.007). Life satisfaction showed inverse associations with depressive symptoms (ρ = −0.391, *p* = 0.003), anxiety symptoms (ρ = −0.425, *p* = 0.001), and loneliness (ρ = −0.372, *p* = 0.006), but was not associated with heart-focused anxiety (ρ = 0.002, *p* = 0.99). Resilient coping (BRCS) was not significantly correlated with any symptom measure (Table 6).

**Table 6:** Spearman correlations between psychosocial variables.

Variable	BRCS	GAD‑7	HAF-17	JSS	PHQ‑9	UCLA‑3	Life satisfaction
BRCS	–	–	–	–	–	–	–
GAD‑7	0.015	–	–	–	–	–	–
HAF-17	0.124	0.466***	–	–	–	–	–
JSS	0.261	0.316*	0.306*	–	–	–	–
PHQ‑9	0.007	0.763***	0.513***	0.431**	–	–	–
UCLA‑3	−0.186	0.365**	0.282*	0.057	0.334*	–	–
Life satisfaction	0.200	−0.425**	0.002	−0.090	−0.391**	−0.372**	–

Spearman’s rho. Higher scores indicate greater symptom burden, except for life satisfaction and BRCS.

*BRCS* Brief Resilient Coping Scale, *GAD‑7* Generalized Anxiety Disorder Scale (7-item), *HAF-17* Heart Anxiety Questionnaire (17-item), *JSS* Jenkins Sleep Scale, *PHQ‑9* Patient Health Questionnaire (9-item), *UCLA‑3* 3-item UCLA Loneliness Scale.

*p < 0.05, **p < 0.01, ***p < 0.001 (two-tailed).

**Fig. 1:**
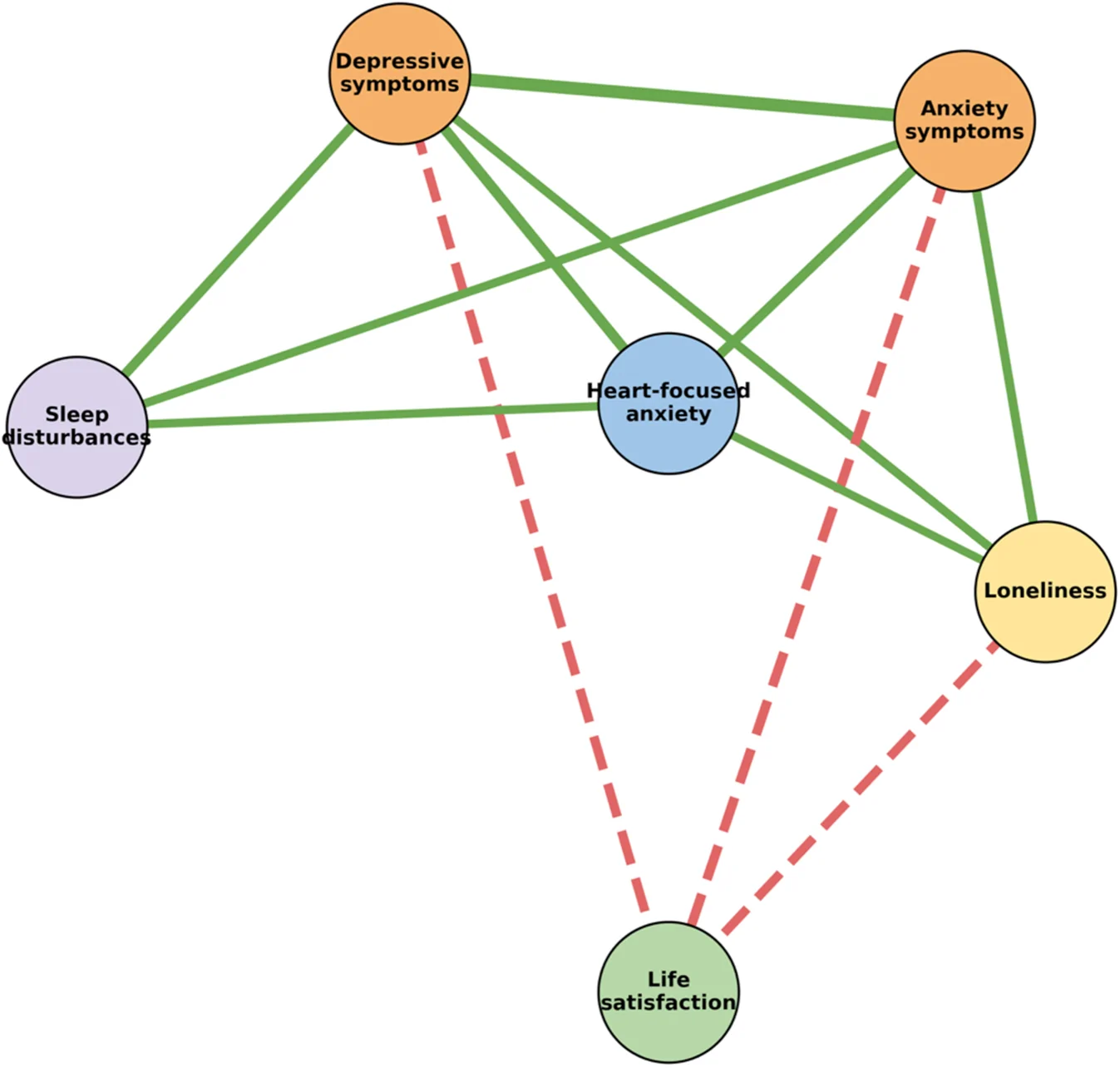
Network of significant psychosocial correlations. Network visualization of statistically significant Spearman correlations (*p* < 0.05) between psychosocial variables. Nodes represent validated questionnaires assessing depressive symptoms (Patient Health Questionnaire, PHQ-9), anxiety symptoms (Generalized Anxiety Disorder Scale, GAD-7), heart-focused anxiety (Heart Anxiety Questionnaire, HAF-17), sleep disturbances (Jenkins Sleep Scale), loneliness (UCLA Loneliness Scale, UCLA-3), and life satisfaction. Solid green edges indicate positive correlations, dashed red edges indicate negative correlations; edge thickness reflects correlation strength

The network visualization illustrates a cluster of significant associations among depressive symptoms, anxiety, sleep disturbances, and loneliness, whereas heart-focused anxiety shows no association with overall life satisfaction (Fig. 1).

## Discussion

In this cross-sectional survey conducted in a routine cardiology care setting, depressive and generalized anxiety symptoms were generally low, whereas heart-focused anxiety as well as stress- and sleep-related complaints were more prominent. In addition, a substantial proportion of patients expressed interest in psychocardiological support, underscoring the practical relevance of systematic psychosocial screening in cardiology. Notably, a substantial subset of patients were not aware of existing psychocardiology services, despite reporting psychosocial burden.

Our findings align with growing evidence that psychological distress is common among patients with cardiovascular disease [16]. Importantly, the present data suggest that limiting screening to depression and generalized anxiety alone may underestimate the broader psychosocial burden experienced by cardiac patients, as a substantial proportion reported at least some perceived stress impairment over the prior 4 weeks (mild impairment in 43.8% and severe impairment in 8.3%). The correlation structure provides further clinically relevant nuance. Depressive and anxiety symptoms were strongly interrelated (ρ = 0.763), reflecting overlapping affective distress. Heart-focused anxiety correlated moderately with both depressive and anxiety symptoms, but was also significantly related to sleep disturbances and loneliness. This pattern suggests that heart-focused anxiety represents a distinct psychosocial dimension clustering with symptom-related domains that are particularly relevant for daily functioning and healthcare utilization, rather than merely reflecting general anxiety.

By contrast, life satisfaction displayed inverse associations with depression, generalized anxiety, and loneliness, but was unrelated to heart-focused anxiety, suggesting that cardiac-specific anxiety may operate as a more situation- and symptom-focused construct, whereas overall life evaluation is more tightly linked to general affective distress and social connectedness. Resilient coping fell in the lower resilient coping range at the group level, yet it was not correlated with any symptom measure in this small sample.

An important implementation-related finding concerns awareness and potential uptake of psychocardiological support. Only 7.0% of respondents reported that they had previously heard of psychocardiology, yet 56.2% indicated at least potential willingness to use such a service (“yes” 8.8%, “maybe” 47.4%). This discrepancy suggests that psychosocial needs do not automatically translate into service utilization and that limited awareness may constitute a relevant barrier to care. Assessing both psychosocial burden and patient awareness of available services may therefore represent a crucial step toward patient-centered implementation of psychocardiological programs.

### Limitations

This study has several strengths. It was conducted in a real-world setting and included a broad range of psychosocial domains using brief, validated instruments. However, several limitations should also be considered. The present findings should be interpreted as preliminary results derived from a relatively small sample in a single-center setting. The cross-sectional design and the small sample size limit the ability to draw conclusions about causality and directions of relationships between psychosocial factors and health outcomes. The use of self-report measures may be influenced by reporting bias and does not replace structured clinical interviews. The anonymous design precluded linkage of psychosocial data with detailed clinical information such as specific cardiac diagnoses, disease severity, cardiac function, comorbidities, or treatment course. Also, the study was conducted at a single tertiary care center, which may limit generalizability to other healthcare settings. In addition, due to the anonymous and non-tracked distribution process, the number of patients approached could not be documented, and response rates could not be calculated. Together with the convenience sampling approach and voluntary participation, this may have introduced selection bias, potentially leading to an under- or overestimation of psychosocial burden. As questionnaires were completed during routine care without a standardized time point, responses may reflect different clinical phases (e.g., acute presentation versus stabilized condition). This may be particularly relevant for heart-focused anxiety, which could be increased during acute or diagnostically uncertain situations. Finally, the sample size precluded robust multivariable analyses and limited subgroup exploration.

Taken together, these preliminary findings suggest that while depressive and generalized anxiety symptoms were on average low, heart-focused anxiety as well as stress- and sleep-related complaints may represent relevant domains of psychosocial burden in routine cardiology care. At the same time, awareness of psychocardiology services was limited, despite substantial patient interest. These findings are in line with current AHA [1] and ESC [16] recommendations for integrating mental health into cardiovascular care and suggest that multidimensional psychosocial screening may help identify clinically relevant but otherwise overlooked aspects of patient burden. Future studies should integrate psychosocial screening data with detailed clinical baseline characteristics, comorbidities, and the course of hospitalization, and examine how such approaches can be effectively linked to tailored interventions and whether this improves patient-reported outcomes and cardiovascular prognosis.

## Data Availability

The datasets generated and/or analyzed during the current study are not publicly available due to the anonymous nature of the survey and data protection regulations but are available from the corresponding author on reasonable request.

## Declarations

All procedures performed in studies involving human participants or on human tissue were in accordance with the ethical standards of the institutional and/or national research committee and with the 1975 Helsinki Declaration and its later amendments or comparable ethical standards. Informed consent was obtained from all individual participants included in the study.
